# Bacteremia Caused by *Mycobacterium wolinskyi*

**DOI:** 10.3201/eid1411.080003

**Published:** 2008-11

**Authors:** Yu-Chuan Chen, Ruwen Jou, Wei-Lun Huang, Shao-Tsung Huang, Keng-Chang Liu, Chorng-Jang Lay, Shu-Mei Chang, Chih-En Tseng, Chun-Liang Lai, Yu-Chieh Su

**Affiliations:** Buddhist Tzu Chi Dalin General Hospital, Chiayi, Taiwan (Y.-C. Chen, K.-C. Liu, C.-J. Lay, S.-M. Chang, C.-E. Tseng, C.-L. Lai, Y.-C. Su); Centers for Disease Control Department of Health, Taipei, Taiwan (R. Jou, W.-L. Huang); Chest Hospital Health Executive Yuan, Tainan, Taiwan (S.-T. Huang); Tzu Chi University School of Medicine, Hualien, Taiwan (K.-C. Liu, C.-J. Lay, S.-M. Chang, C.-E. Tseng, C.-L. Lai, Y.-C. Su)

**Keywords:** Bacteremia, Mycobacterium wolinskyi, lymphoma, rituximab, letter

**To the Editor:** *Mycobacterium wolinskyi* is a rapidly growing mycobacterium that belongs to the *M. smegmatis* group, which includes *M. smegmatis* sensu stricto and 2 species described in 1999 (*M. goodii* and *M. wolinskyi*) (*1*). Only 9 cases of infection caused by *M.* *wolinskyi* have been reported (*1**–**3*), and these included 3 cases of bone infection and 1 case of infection of a hip prosthesis. All patients had a history of surgery after traumatic injury and all specimens were isolated from the surgical wound. In our study, we used molecular diagnostic tools and report a case of bacteremia caused by *M. wolinskyi*.

In November 2006, we diagnosed non-Hodgkin lymphoma in a 22-year-old woman. A venous port was implanted, and 4 courses of rituximab (anti-CD20 monoclonal antibody) plus additional chemotherapy (cyclophosphamide, epirubicin, vicristine and prednisolone) were administered from December 2006 through May 2007. No unfavorable sequelae occurred after chemotherapy, and the tumor showed a complete response. In August 2007, we admitted the patient to our hospital because of a spiking high fever (up to 40°C), chills, and pain in the left knee. On physical examination, the patient had a tender, warm, erythematous, and swollen left knee. These symptoms progressed to other joints, including the left hip and ankle.

Laboratory data showed a normal leukocyte count (3.4 × 10^9^ cells/L). The patient’s C-reactive protein level increased from 1.13 mg/dL (on the day of admission) to 24.95 mg/dL (7 days after admission). We drew 2 sets of blood samples from a peripheral vein for culture and incubated these cultures (BACTEC 9240 Continuous Monitoring Blood Culture System; Becton Dickinson, Sparks, MD, USA) using BACTEC Aerobic Plus and Anaerobic Plus medium (Becton Dickinson). Within 3 days, the cultures tested positive for acid-fast bacilli.

The isolate was identified by 16S rRNA gene amplification of an 880-bp region (corresponding to positions 27–907), as previously described (*4**,**5*). For amplification, we used broad-range primers 16S-27f (5′-AGA GTT TGA TCM TGG CTC AG-3′) and 16S-907r (5′-CCG TCA ATT CMT TTR AGT TT-3′). For sequencing 16S rDNA, we used either the primer 16S-27f or 16S-519r (5′-GWA TTA CCG CGG CKG CTG-3′). We performed both forward and reverse (5′ and 3′) sequencing. For accurate analysis of the data, a 492-bp variable region (corresponding to positions 27– 519) was carefully analyzed after it was compared with sequences of *Mycobacterium* spp. in the BLAST database (www.ncbi.nlm.nih.gov), as described (*6*). The results showed 99% similarity between our isolate and *M. wolinskyi*.

A few days later, we obtained synovial fluid by needle biopsy and cultured samples in BACTEC Aerobic Plus and Anaerobic Plus medium (Becton Dickinson) and on trypticase soy agar. Within 3 days, these cultures were also positive for *M. wolinskyi*. Arthroscopically assisted arthrocentesis and debridement showed a turbid joint and the debrided tissue showed inflammatory processes within the synovial tissue and the presence of acid-fast bacilli (Figure). We grew cultures of acid-fast bacilli on trypticase soy agar after 2 to 4 days. The colonies were nonchromogenic, smooth to mucoid, and off-white to cream on Middlebrook 7H10 and trypticase soy agar.

**Figure:**
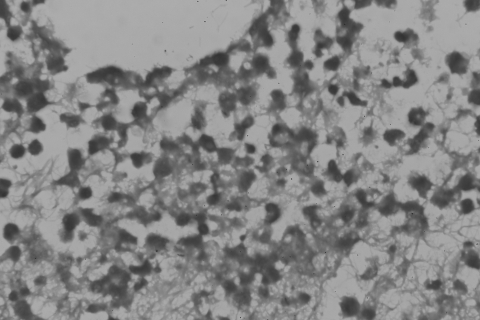
Histologic image of debrided tissue of the patient, showing inflammatory processes within the synovial tissue and the presence of an acid-fast bacillus (magnification ×400, acid-fast stain).

We tested the in vitro antimicrobial susceptibility using the broth dilution method (*7*). The isolate susceptible to amikacin, cefoxitin, imipenem, doxycycline, and ciprofloxacin and resistant to sulfamethoxazole, clarithromycin, and tobramycin. We initiated treatment of the patient with moxifloxacin, minocycline, and amikacin 1 day after the athroscopy and the patient’s fever subsided within 72 hours. We continued amikacin therapy for 1 month and administered moxifloxacin and minocycline for 6 months.

This patient is unique because she had a case of bacteremia caused by *M. wolinskyi,* and she had no history of major traumatic injury. The bacterium might have been introduced during implantation of the venous port or during minor trauma that went unnoticed. The chemotherapeutic regimen administered to our patient may have played a role in the infection. Immunosuppression by treatment with rituximab (an anti-CD20 monoclonal antibody) and a steroid during chemotherapy may have worsened the patient’s B-cell function and thereby weakened her immunity*.* Surgical debridement followed by antimicrobial therapy for at least 6 months is the suggested treatment for *M. wolinskyi* infection, and we followed this regimen. Because of the frequency of relapse and resistance, we used combination therapy with multiple antimicrobial agents.

This case suggests that immunocompromised patients may be vulnerable to infection by rapidly growing mycobacterium such as *M. wolinskyi*. In such cases, we suggest antimicrobial drug treatment, based on in vitro susceptibility. More data on antimicrobial drug susceptibility should be collected for treatment of this type of infection.
